# The Maize ZmBES1/BZR1-9 Transcription Factor Accelerates Flowering in Transgenic *Arabidopsis* and Rice

**DOI:** 10.3390/plants12162995

**Published:** 2023-08-19

**Authors:** Yuan Liu, Hongwanjun Zhang, Wenqi Feng, Xiaolong Lin, Aijun Gao, Yang Cao, Qingqing Yang, Yingge Wang, Wanchen Li, Fengling Fu, Haoqiang Yu

**Affiliations:** Key Laboratory of Biology and Genetic Improvement of Maize in Southwest Region; Maize Research Institute, Sichuan Agricultural University, Chengdu 611130, China

**Keywords:** ectopic expression, BES1/BZR1, vegetative growth, regulation, flowering period

## Abstract

In model plants, the BRI1-EMS suppressor 1 (BES1)/brassinazole-resistant 1 (BZR1) transcription factors play vital roles in regulating growth, development, and stimuli response. However, the roles of maize *ZmBES1/BZR1* members are largely unknown. In this research, the *ZmBES1/BZR1-9* gene was ectopically expressed in *Arabidopsis* and rice for the phenotyping of flowering. We found that the complementation and overexpression of *ZmBES1/BZR1-9* in *bes1-D* mutant and wild type *Arabidopsis* both resulted in early flowering that was about 10 days shorter than in the untransformed control under long-day conditions. In addition, there was no difference in the rosette leaf number between all transgenic lines and the control. Subsequently, the *ZmBES1/BZR1-9* gene was overexpressed in rice. It was found that overexpression lines of rice exhibited early flowering with heading dates that were 8 days shorter compared with untransformed plants. Moreover, the results of RNA-seq and qRT-PCR showed that five flowering-regulated genes, namely *At2-MMP*, *AtPCC1*, *AtMYB56*, *AtPELPK1*, and *AtPRP10,* were significantly up-regulated in all complementary and overexpressing lines of *Arabidopsis*. Meanwhile, the results of RNA-seq showed that 69 and 33 differentially expressed genes (DEGs) were up- and down-regulated in transgenic rice, respectively. Four flowering-related genes, namely *OsGA20OX1*, *OsCCR19*, *OsBTBN19*, and *OsRNS4* were significantly up-regulated in transgenic lines. To sum up, our findings demonstrate that *ZmBES1/BZR1-9* is involved in controlling flowering and provide insights into further underlying roles of *BES1/BZR1s* in regulating growth and development in crops.

## 1. Introduction

In higher plants, flowering is an important developmental process and marks the transition from vegetative growth to reproductive growth [1]. The photoperiod, vernalization, autonomic, gibberellin, age, and temperature pathways are among the main flowering-regulatory cues in plants. But, as crucial steroidal hormones, brassinosteroids (BRs) have been confirmed to control various biological processes in plants, such as growth and development regulation as well as stress resistance [2,3]. The BR signal is perceived by the receptor BRI1 and the co-receptor BAK1 on the cell membrane and triggers the phosphorylation and dephosphorylation of various downstream proteins, including BSKs (BR SIGNALING KINASES), CDGl (CONSTITUTIVE DIFFERENTIAL GROWTH1F), BSU1 (BRI1-SUPPRESSORF), BIN2 (BR-INSENSITIVE 2) [4,5,6,7]. Importantly, the inactivated BIN2 cannot phosphorylate the BES1 (BRI1-EMS-SUPPRESSOR 1) and BZR1 (BRASSINAZOLE RESISTANT 1) transcription factors, which also can be dephosphorylated by PP2A [6,8,9]. Thereafter, the activated BES1/BZR1s accumulate in the nucleus and regulate the transcription of nuclear genes by directly binding to elements of the *E-box* (CANNTG) or *BRREs* (CGTGT/CG) in gene promoters to regulate plant growth and BR synthesis [10,11,12,13].

As core transcription factors, previous studies have shown that BZR1/BES1s not only meditate BR signaling but also cross-talk with other hormone signals to regulate plant growth and stress resistance [14,15]. For instance, BES1/BZR1 can inhibit BR synthesis by binding to the BR synthesis genes promoter of *DWF4* and *CPD* to inhibit their expression [10,12,16]. BES1 hinders ABI5 (ABSCISIC ACID INSENSITIVE5) to promote seed germination via ABA signaling and involves light to regulate plant growth mediated by the SINAT E3 ligase [17,18]. Exogenous GA can activate the BZR1-mediated BR signaling pathway [15]. Likewise, BES1/BZR1s regulate the adaptation to freezing, heat, phosphate-deficient, drought, and salt stress by up-regulating the expression of multiple stress-related genes in *Arabidopsis*, wheat, maize, tomato, and apple [19,20,21,22,23,24,25,26,27,28].

Moreover, BES1/BZR1s also recruit other regulators to regulate plant developmental processes. In *Arabidopsis*, dephosphorylated BES1 interacts with the UV receptor UVR8 and the blue light receptors CRY1 and CRY2 to regulate plant photomorphogenesis by inhibiting the binding between BES1 and DNA [29]. BES1 interacts with ERF6 (Early flowering 6) and its homolog REF6 to regulate target gene expression and to control flowering time [30]. BZR1 interacts with the cyclophilin Cyp20-2 to co-regulate flowering by suppressing *FLD* (*FLOWERING LOCUS D*) expression [31]. BZR1, together with BES1-INTERACTING MYC-like protein (BIM), recruits the H3K27 (histone 3 lysine 27) demethylase to activate *FLC* expression, leading to the inhibition of the flower transition [32]. Recently, it has been reported that BES1 acts on BEE1 (BR ENHANCED EXPRESSION 1), which directly binds to *FT* (*FLOWERING LOCUS T*), to control photoperiodic flowering [33]. In rice, OsBZR1 targets and interacts with histone deacetylase HDA703 to control rice growth and the heading date by repressing *Ghd7* (*HEADING DATE 7*) expression [34]. However, the function of BES1/BZR1s is largely unknown in crops.

In maize, a total of eleven *ZmBES1/BZR1* genes were identified in the maize genome in our previous study and by another group [35,36]. Subsequently, we confirmed that *ZmBES1/BZR1-3* and *-9* negatively regulates drought tolerance, while *ZmBES1/BZR1-5* positively regulates salt and drought tolerance, the ABA response, and seed development [21,25,37]. Furthermore, *ZmBES1/BZR1-2* has also been proven to positively regulate seed size and to promote the enlargement of the cotyledon and rosette leaves [38]. These reports suggest that *ZmBES1/BZR1s* members act on plant growth and development with functional diversity. However, whether *ZmBES1/BZR1s* is involved in flowering regulation is unknown. In this study, the function of *ZmBES1/BZR1-9* in regulating flowering was evaluated by the heterologous expression of *ZmBES1/BZR1-9* in *Arabidopsis* and rice. The study proves that *ZmBES1/BZR1-9* is an activator of flowering and will provide insights into further underlying roles of BES1/BZR1s in regulating growth and development in crops.

## 2. Results

### 2.1. Generation of Transgenic Lines

**Figure 1:**
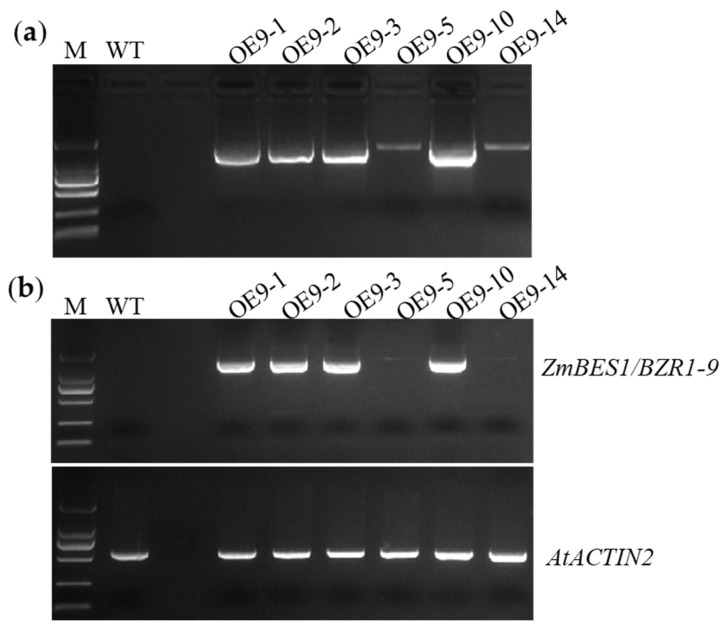
Identification of transgenic *Arabidopsis* lines. (**a**) PCR detection. (**b**) RT-PCR. OE9-1, 9-2, 9-3, 9-5, 9-10, and 9-14 represent homozygous lines overexpressing the *ZmBES1/BZR1-9* gene in *Arabidopsis*. The 911 bp fragment of *ZmBES1/BZR1-9* was amplified and detected by PCR (**a**) and RT-PCR (**b**), respectively. M, DNA 2000 standard consisting of a 2000, 1000, 750, 500, 250, and 100 bp ladder from top to bottom. WT, wild type. The 545 bp fragment of *AtACTIN2* was amplified and used as a reference.

**Figure 2:**
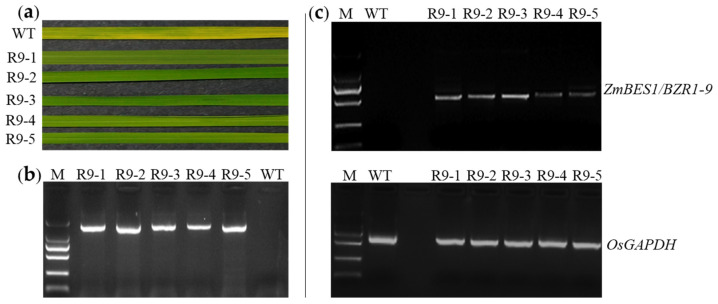
Screening of transgenic rice. (**a**) Antibiotic screening. (**b**) PCR amplification. (**c**) RT-PCR. R9-1, R9-2, R9-3, R9-4, and R9-5 mean transgenic rice lines expressing the *ZmBES1/BZR1-9* gene. The 911 bp and 582 bp fragments of *ZmBES1/BZR1-9* were amplified and detected by PCR and RT-PCR, respectively. M, DNA 2000 standard consisting of a 2000, 1000, 750, 500, 250, and 100 bp ladder from top to bottom. WT, wild type. The 629 bp fragment of the *OsGAPDH* gene was amplified and used as a reference.

To identify the function of *ZmBES1/BZR1-9*, it was transformed into *Arabidopsis* and rice, respectively. The positive transformants of *Arabidopsis* showed normal growth and robust green plants on plates with 50 mg/L kanamycin. In total, six homozygous lines were generated as identified by PCR amplification and reverse transcription PCR (RT-PCR), and these were named OE9-1, 9-2, 9-3, 9-5, 9-10, and 9-14. In these lines, the *ZmBES1/BZR1-9* gene was successfully amplified from their genomic DNA (gDNA) and cDNA, respectively. However, there was no amplicon in the wild type (WT) (Figure 1). Meanwhile, five homozygous lines of rice expressing *ZmBES1/BZR1-9* were produced and defined as R9-1, 9-2, 9-3, 9-4, and 9-5. These lines exhibited green leaves after soaking in 100 mg/L hygromycin solution, and the insertion of *ZmBES1/BZR1-9* in the rice genome was confirmed by PCR and RT-PCR, respectively (Figure 2). The complementary lines L9-3 and L9-5 of the *Arabidopsis* mutant were produced in our previous study [21]. Hence, the complementary *Arabidopsis* lines L9-3 and 9-5, the overexpressing *Arabidopsis* lines OE9-2 and OE9-3, as well as the overexpressing rice lines R9-1 and R9-5 were used for phenotyping.

### 2.2. Expression of ZmBES1/BZR1-9 Accelerates Flowering in Arabidopsis

**Figure 3:**
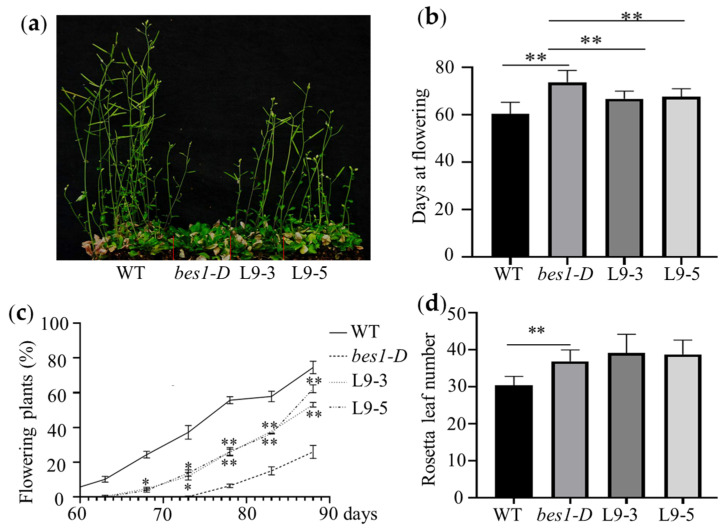
The phenotype of complementary lines under LD conditions (14 h light/10 h dark). (**a**) Flowering phenotype. (**b**) Days at flowering. (**c**) Dynamic statistics of flowering time. (**d**) The number of rosette leaves. The seeds of each line were germinated and grown in growth chambers under LD conditions. The days from germination to flowering, the percentage of flowering plants over the same time period, and the total rosette leaf number were measured from 25 plants in each replicate. WT, wild type. *bes1-D*, untransformed mutant. L9-3 and L9-5 represent complementary lines of *ZmBES1/BZR1-9* in the *bes1-D* mutant. * and ** represent *p* < 0.05 and *p* < 0.01, respectively.

To explore the function of *ZmBES1/BZR1-9* in the floral transition, the days from germination to flowering (DTF) of the complementary lines L9-3 and L9-5 were measured. As shown in Figure 3, under long-day (LD) conditions, the L9-3 and L9-5 lines exhibited early flowering and a significantly higher rate of flowering plants in the same growth stage compared with the *bes1-D* mutant, which showed delayed flowering compared with the WT. However, there was no difference in the total rosette leaf number (RLN) between the *bes1-D*, L9-3, and L9-5 lines. Meanwhile, the RLN of *bes1-D* was significantly higher than that of WT.

**Figure 4:**
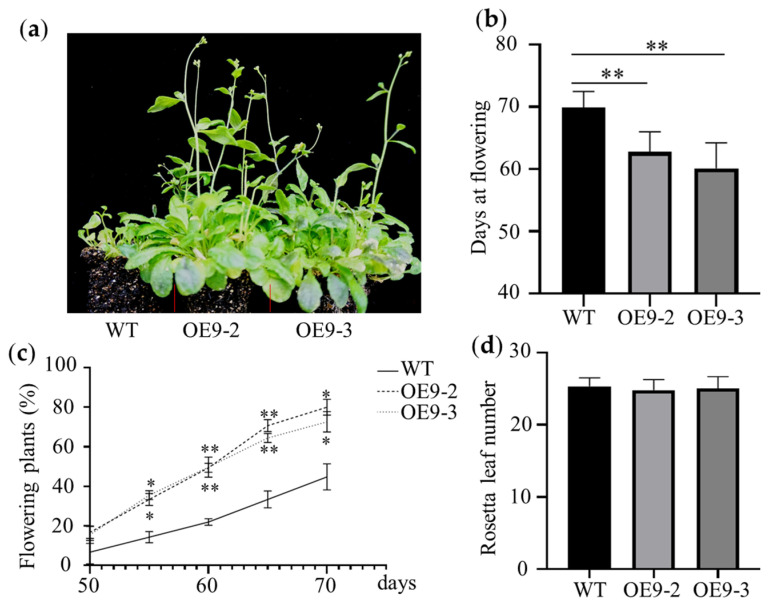
The phenotype of overexpressed lines under LD conditions (14 h light/10 h dark). (**a**) Flowering phenotype. (**b**) Days at flowering. (**c**) Dynamic statistics of flowering time. (**d**) The number of rosette leaves. The seeds of each line were germinated and grown in growth chambers under LD conditions. The days from germination to flowering, the percentage of flowering plants over the same time period, and the total rosette leaf number were measured from 25 plants in each replicate. WT, wild type. OE9-2 and OE9-3 represent lines overexpressing *ZmBES1/BZR1-9*. * and ** represent *p* < 0.05 and *p* < 0.01, respectively.

Meanwhile, to validate the early-flowering phenotype regulated by *ZmBES1/BZR1-9*, we generated the overexpressed lines OE9-2 and OE9-3 and conducted a phenotype characterization as a complementary assay. Similarly, early flowering was also observed in overexpressing lines under LD conditions. The DTF of the OE9-2 and OE9-3 lines was about 10 days earlier than that of WT. Compared with WT, the number of flowering plants in OE9-2 and OE9-3 was significantly higher from 55 days after sowing (Figure 4). At the same time, there was also no significant difference in RLN among different lines.

The above results indicate that the heterologous expression of the *ZmBES1/BZR1-9* gene accelerates floral transition but does not affect the vegetative growth in *Arabidopsis*.

**Figure 5:**
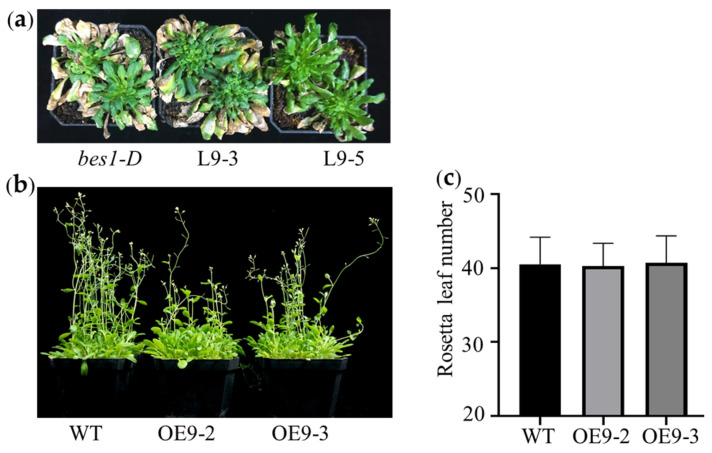
The flowering phenotype of transgenic *Arabidopsis* under SD conditions. (**a**) The phenotype of complementary lines. (**b**) The phenotype of overexpressed lines. (**c**) The number of rosette leaves. L9-3 and L9-5 represent complementary lines. OE9-2 and OE9-3 represent overexpressing lines. *bes1-D*, mutant; WT, wild type.

Moreover, in order to determine whether ZmBES1/BZR1-9 promotes flowering via the photoperiod pathway, L9-3, L9-5, and bes1-D as well as OE9-2, OE9-3 and WT were cultured under short-day (SD) conditions for phenotyping. We found that L9-3, L9-5, and bes1-D showed no bolting, with a severe vegetative growth phenotype at 125 days after sowing under SD conditions (Figure 5a). Likewise, although OE9-2, OE9-3, and WT gradually blossomed after 95 days of planting, there was no difference in flowering time among these lines (Figure 5b). The RLN also showed no difference (Figure 5c).

### 2.3. ZmBES1/BZR1-9 Promotes the Expression of Flowering-Related Genes in Arabidopsis

**Figure 6:**
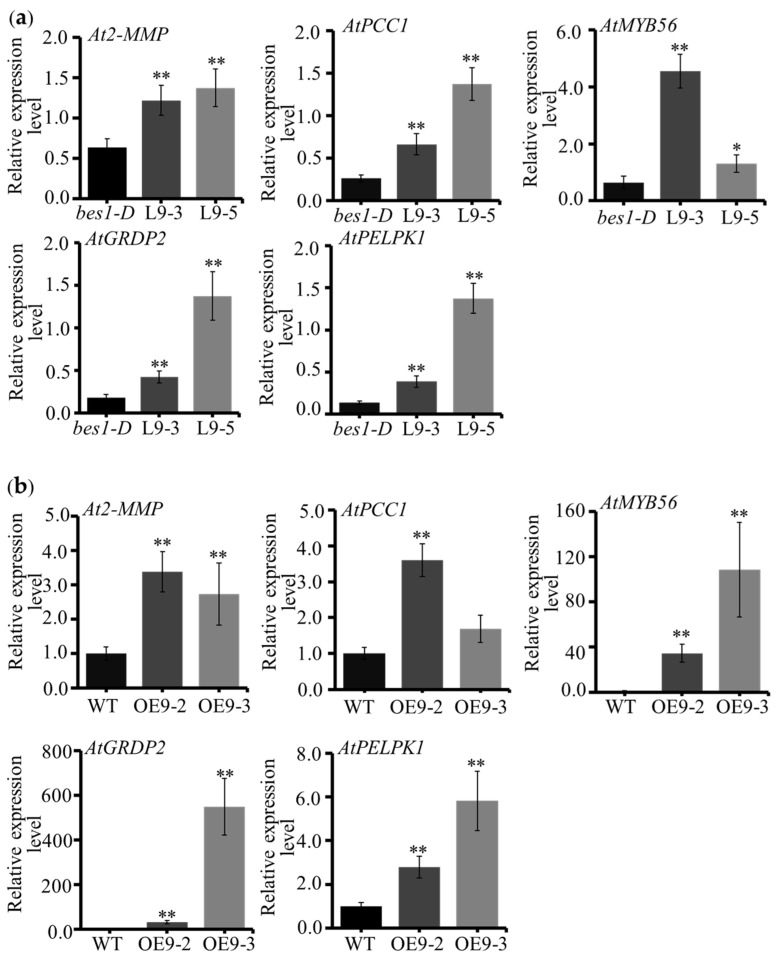
The relative expression of flowering-related genes in complementary lines (**a**) and overexpressing lines (**b**). L9-3 and L9-5 represent complementary lines; OE9-2 and OE9-3 represent overexpressing lines. *bes1-D*, mutant; WT, wild type. *AtACTIN2* was used as a reference. * and ** represent *p* < 0.05 and *p* < 0.01, respectively.

RNA-sequencing (RNA-seq) was performed in our previous study to interrogate the transcriptomic changes caused by *ZmBES1/BZR1-9* in the L9-3 and L9-5 lines [21]. To investigate the potential mechanism of *ZmBES1/BZR1-9* in controlling flowering in transgenic lines, the differentially expressed genes (DEGs) related to flowering were explored. In comparison with the *bes1-D* mutant, five DEGs associated with the regulation of flowering were identified in both the L9-3 and L9-5 lines, namely *At2-MMP* (AT1G70170), *AtPCC1* (AT3G22231), *AtMYB56* (AT5G17800), *AtGRDP2* (AT4G37900), and *AtPELPK1* (AT5G09530). Therefore, quantitative real-time PCR (RT-qPCR) was performed to analyze the expression of these genes in complementary lines, overexpressed lines, and an untransformed control. As shown in Figure 6, the expression of *At2-MMP*, *AtPCC1*, *AtMYB56*, *AtGRDP2*, and *AtPELPK1* genes was significantly up-regulated in L9-3 and L9-5, as well as in OE9-2 and OE9-3, compared with *bes1-D* and WT, respectively. Meanwhile, 8, 10, 8, 13, and 16 *E-box* elements were found in the *At2-MMP*, *AtPCC1*, *AtMYB56*, *AtGRDP2*, and *AtPELPK1* promoter regions, respectively (Table S2). The result indicates that *ZmBES1/BZR1-9* promotes the flowering of transgenic *Arabidopsis* by up-regulating the expression of these genes.

### 2.4. ZmBES1/BZR1-9 Accelerates Flowering in Rice

**Figure 7:**
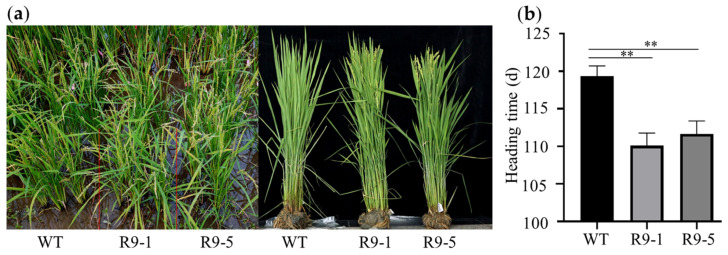
The flowering phenotype of transgenic rice under SD conditions. (**a**) Phenotype. (**b**) Statistics of heading date. The transgenic rice plants were grown in Sanya in winter from mid-Oct to mid-March under natural SD conditions with 9.5–12h light. The values represent means ± SEs from three biological replicates. R9-1 and R9-5 represent transgenic lines. WT, wild type. ** represents *p* < 0.01.

To further analyze the role of *ZmBES1/BZR1-9* in regulating flowering, the transgenic rice lines R9-1, R9-5, and the WT line were grown in Chengdu in summer (LD) and in Sanya in winter (SD). The results of phenotyping showed that there was no difference in heading date between the transgenic lines and the WT under LD conditions in summer. In contrast, compared with the WT, the transgenic lines R9-1 and R9-5 exhibited an earlier heading date and their flowering time was shortened by about 8 days under SD conditions in winter (Figure 7).

### 2.5. ZmBES1/BZR1-9 Regulates the Expression of Flowering-Associated GENES in Rice

**Figure 8:**
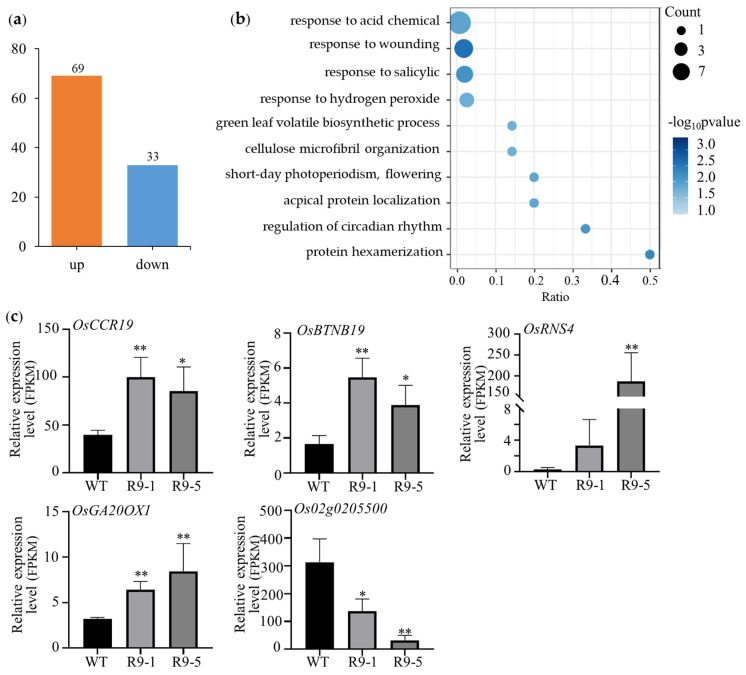
DEGs of transgenic rice with *ZmBES1/BZR1-9* gene compared with WT. (**a**) The common DEGs shared by R9−1 and R9−5. (**b**) GO analysis of common DEGs. (**c**) The relative expression level of flowering-related genes. R9−1 and R9−5 represent transgenic lines. WT, wild type. * and ** represent *p* < 0.05 and *p* < 0.01, respectively.

RNA-seq was also conducted to analyze the DEGs regulated by ZmBES1/BZR1-9 in transgenic rice. As shown in Figure 8, compared with WT, a total of 102 common DEGs were identified and shared by the R9-1 and R9-5 lines. Among them, 67.65% of DEGs (69) were up-regulated, and 32.35% of DEGs (33) were down-regulated in the two transgenic lines. Gene Ontology (GO) analysis suggested that five DEGs were associated with flowering and involved in short-day photoperiodism and circadian rhythm; these were *OsGA20OX1* (Os03g0856700), *OsCCR19* (Os09g0419200), *OsBTBN19* (Os09g0420900), *OsRNS4* (Os09g0537700), and an unknown gene (Os02g0205500). Similarly, 15, 8, 14, 13, and 8 *E-box* elements were also found in the *OsGA20OX1*, *OsCCR19*, *OsBTBN19*, *OsRNS4*, and *Os02g0205500* gene promoters, respectively (Table S2).

## 3. Discussion

Previous studies show that BR signaling is an important pathway involved in plant-flowering regulation. For instance, BR-insensitive and deficient mutants exhibit delayed flowering morphology, confirming that the components of the BR signal control the flowering transition [30,31,39,40,41]. BES1/BZR1s are key hubs in the BR signal [10,13]. In the present study, we found that the complementation and overexpression of the *ZmBES1/BZR1-9* gene in WT and mutant *Arabidopsis* resulted in early flowering under LD conditions compared with the control (Figure 3 and Figure 4). Nevertheless, there was no significant difference in flowering time between the transgenic *Arabidopsis* and the control, which both exhibited exuberant vegetative growth of each line under SD conditions (Figure 5). The RLN is a key indicator of *Arabidopsis* flowering [42], but the transgenic plants showed no difference in RLN under LD and SD conditions (Figure 5). Meanwhile, transgenic rice exhibited a shorter heading date and about 8 days earlier flowering than WT under SD conditions, but there was no difference under natural LD conditions (Figure 7). This indicates that the *ZmBES1/BZR1-9* gene positively regulates flowering but does not affect the vegetative growth process.

It has been confirmed that BES1 acts as a positive regulator of photoperiodic flowering in *Arabidopsis* [33]. As previously reported, the overexpression of BES1 promotes flowering, and its mutant and RNAi plants delayed flowering and possess significantly higher RLN than WT under LD conditions [32,33,43]. Similarly, overexpression of *MiRZFP34* resulted in early flowering in transgenic *Arabidopsis* but no difference in RLN was observed [44]. Likewise, it was previously proven that histone deacetylase HDA703 interacts with OsBZR1 to control rice growth and heading by inhibiting *Ghd7* expression [34]. Hence, it is speculated that the different phenotypes of transgenic *Arabidopsis* and rice expressing *ZmBES1/BZR1-9* under LD and SD conditions may be due to the *Arabidopsis* being an LD plant but the rice being an SD plant [42]. In *Arabidopsis*, under LD conditions, the CO (CONSTANS) protein accumulated and induced the expression of *FT* and its homolog *TSF* (*TWIN SISTER OF FT*) to promote flowering [45,46]. However, the CO-homologous Hd1 promotes early heading by up-regulating the expression of the *FT*-homologous *Hd3a* gene under SD conditions in rice [47,48]. Therefore, we speculate that the *ZmBES1/BZR1-9* gene regulates flowering through different photoperiod-mediated pathways in *Arabidopsis* and rice.

Furthermore, we showed that ZmBES1/BZR1-9 up-regulated the expression of *At2-MMP*, *AtPCC1*, *AtMYB56*, *AtGRDP2*, and *AtPELPK1*, as well as of *OsGA20OX1*, *OsCCR19*, *OsBTBN19*, and *OsRNS4*, in transgenic *Arabidopsis* and rice, respectively (Figure 6 and Figure 8). This can be explained by the binding of ZmBES1/BZR1-9 to *E-box* elements in these genes promoters to promote their expression (Table S2) because the ZmBES1/BZR1-9 protein localizes in the nucleus and functions as a transcription factor [21]. It was confirmed that BES1/BZR1s can directly bind to *E-box* or *BRRE* elements to regulate the transcription of target genes [10,13]. Previous studies confirmed that *at2-mmp* mutant, *AtPCC1* and *AtPELPK1* RNAi plants, and *AtGRDP2* knockout lines showed delayed flowering, suggesting their positive roles in regulating flowering [49,50,51,52]. Likewise, *PPC1* exhibits a circadian-regulated expression pattern, is involved in light-regulated development via interaction with the COP9 signalosome subunit 5, and regulates the flowering transition in the photoperiod-dependent pathway [52,53,54]. *AtGRDP2* also regulates female gametophyte development via the auxin pathway, and plants overexpressing it show early flowering [50,55]. Meanwhile, BES1 directly represses *AtMYB56*, which positively regulates the quiescent center and cell division [56,57]. In rice, *OsGA20OX1* influences GA levels, is expressed in reproductive meristems, and crosstalks with cytokinin to regulate growth and development [58,59]. GA also regulates flowering [60,61]. *OsRNS4* is regulated by phytochrome (pyh) A-, B-, and C-mediated light signals in rice [62]. It has been shown that phyA, B, and C play crucial roles in plant flowering [63,64,65,66]. In addition, *OsBTBN19* and its homolog, *NPY1*, encode BTB domain proteins, which are reported to regulate flowering [67,68], although the *OsBTBN19* and *OsCCR19* genes were not directly confirmed to regulate flowering.

The study suggests that the *ZmBES1/BZR1-9* gene positively regulates flowering in transgenic *Arabidopsis* and rice and that its overexpression can be used to shorten the flowering period. Meanwhile, the mechanism of *ZmBES1/BZR1-9* in regulating flowering in maize is unknown and will be revealed in our future studies.

## 4. Materials and Methods

### 4.1. Plants Materials and Growth Conditions

*Arabidopsis thaliana* (Col-0) and *Oryza sativa* (Nipponbare) were used for the overexpression of the *ZmBES1/BZR1-9* gene. The complementary lines (L9-3 and L9-5) of the *Arabidopsis bes1-D* mutant were previously produced in our laboratory [21]. All *Arabidopsis* plants were grown in the growth chambers under an artificial LD photoperiod (14 h light/10 h dark, LD) or SD photoperiod (10 h light/14 h dark, SD) under 60–70% relative humidity at 22 °C. The rice seedlings were grown in the field in Chengdu in summer (from mid-May to mid-July; LD conditions with 13.4–14 h of light) and in Sanya in winter (from mid-Oct to mid-next March; natural SD conditions with 9.5–12 h light).

### 4.2. Vector Construction and Transformation

The specific primers 1300-F and 1300-R (Table S1) were designed using Primer5.0, synthesized at Sangon Biotech (Shanghai, China), and used to amplify the coding sequence (CDS) of the *ZmBES1/BZR1-9* gene from the *35S::ZmBES1/BZR1-9-eGFP* plasmid constructed previously [21]. The PCR products and the pCAMBIA1300 plasmid were digested using *Hind* III and *BamH* I. After digestion, the PCR products were subsequently cloned into *Hind* III and *BamH* I sites of the pCAMBIA1300 plasmid to generate *35S::ZmBES1/BZR1-9* using the ClonExpress II One Step Cloning Kit (Vazyme, Nanjing, China). The *ZmBES1/BZR1-9* gene was driven by the *35S* promoter and terminated by CaMV Poly(A).

The *35S::ZmBES1/BZR1-9* plasmids were introduced into the *Agrobacterium tumefaciens* strain GV3101 for the next transformation. To create overexpressed lines of *Arabidopsis*, the transformation of *Arabidopsis* and rice was performed by the floral dip method and *Agrobacterium*-mediated calli transformation, respectively [69,70]. After transformation, the seeds of transgenic *Arabidopsis* were screened using 50 mg/L kanamycin (Sigma, St. Louis, MI, USA) to screening for transformants. The transgenic rice plants were screened by using 100 ng/mL hygromycin B (Coolaber, Beijing, China). The positive *Arabidopsis* seedlings with kanamycin resistance and positive rice seedlings with hygromycin resistance were harvested individually. In the T_2_ generation, the plants showed a 3:1 segregation for resistance/susceptibility to kanamycin or hygromycin and were self-pollinated to generate T_3_. Then, the seeds of each line were screened using the same methods. The homozygous lines were identified without segregation and used in the next study.

### 4.3. PCR and RT-PCR

The gDNA of the transgenic lines and the WT line was extracted using the CTAB method [71]. To confirm the insertion of *ZmBES1/BZR1-9* in the genome of *Arabidopsis* and rice, a pair of specific primers, 9F and 9R, were designed, synthesized, and used to amplify a 911 bp fragment from *ZmBES1/BZR1-9*. Moreover, the total RNA of each line was extracted, and the gDNA was removed and reverse-transcribed to cDNA using the RNAiso Plus kit (Takara) and HiScript^®^ II 1st Strand cDNA Synthesis Kit (+gDNA wiper), respectively. The RT-PCR was performed to detect the transcription of the *ZmBES1/BZR1-9* gene in transgenic lines. Meanwhile, the primer pairs qAf/qAr and qGf/qGr were designed, synthesized, and used to amplify *AtACTIN2* and *OsGAPDH* genes, respectively, which were used as the internal controls. The sequences of primers used for RT-PCR are also listed in Table S1.

### 4.4. Phenotyping of Transgenic Lines

To analyze the flowering time of transgenic *Arabidopsis*, the seeds of the complementary lines, overexpressed lines, WT, and *bes1-D* mutant were sown in soil and cultured in the growth chambers under the conditions described above. The days from germination to flowering, the total rosette leaf number, and the percentage of flowering plants over the same time period were measured from 25 plants and used to monitor the flowering time, as described by Li et al. [32] and Wang et al. [33]. For the phenotyping of transgenic rice, the days from seed germination to the appearance of the first main panicle were counted and used to detect the heading date, as described by Lu et al. [44]. In each replicate, 20 plants of every line were scored. Three replicates of each experiment were performed in this study. All statistical data were calculated using GraphPad Prism and Microsoft Excel 2017 and were presented as the mean ± SE. Student’s *t*-tests were used to analyze the significance of the data between the transgenic lines and the WT. * and ** represent *p* < 0.05 and <0.01, respectively.

### 4.5. RNA-Seq Analysis

The RNA-seq analysis was conducted as our previous study [21]. In brief, the total RNA was extracted from two-week-old seedlings of R9-1, R9-2, and WT using the RNAprep Pure Plant Kit. Then, each RNA sample was qualified by testing their quality and integrity and used for sequencing library preparation using the Bioanalyzer 2100 and the VAHTSTM mRNA-seq V2 Library Prep Kit, respectively. The library was sequenced at the Sanshu Biotechnology Company (Shanghai, China) using the Novaseq 6000 system. The sequencing data were analyzed as described by Sun et al. [25]. The raw data were evaluated and filtered by removing sequencing adapters and low-quality reads as well as contaminants to produce clean data using FastQC (version 0.11.2) and Trimmomatic (version 0.36). Then, clean data were aligned with the *Arabidopsis* genome (TAIR 10) using Hisat2 and used to assemble transcripts of each gene using StringTie. The read counts of each gene assembled by StringTie were used to identify DEGs using DESeq2 with a *p*-value < 0.05 and |FoldChange| > 2. The GO enrichment analysis was performed using KOBAS accessed on 15 February 2023 (http://kobas.cbi.pku.edu.cn/anno_iden.php).

### 4.6. qRT-PCR

The expression of candidate genes in transgenic lines was analyzed by qRT-PCR using PerfectStart^®^Green qPCR SuperMix (TransGen, Beijing, China) in the CFX96^TM^ Real-Time System (Bio-Rad, Hercules, CA, USA), as described in our previous study [25]. The procedure of qRT-PCR consisted of a two-step temperature cycle with pre-degeneration at 95 ℃ for 30 s, 39 cycles of degeneration at 95 for 5 s, and an extension step at 58 °C for 30 s. The temperature was set to increase to 95 ℃ by 0.5 ℃/s at the end of each last cycle to differentiate between specific and non-specific amplicons. Likewise, the *AtACTIN2* and *OsGAPDH* genes were amplified using the primer pairs qAf/qAr and qGf/qGr, respectively, and used as the internal controls. The relative expression level was normalized following the 2^−ΔΔCt^ method [72]. The sequences of these candidates were derived from the National Center for Biotechnology Information (NCBI) or the *Arabidopsis* Information Resource (TAIR) and used to design primers using primer-BLAST accessed on 5 November 2021 (https://www.ncbi.nlm.nih.gov/tools/primer-blast/index.cgi?LINK_LOC=BlastHome). The primers are listed in Table S1. The candidate genes include *AtACTIN2* (AT3G18780), *OsGAPDH* (Os04g40950), *At2-MMP* (AT1G70170), *AtPCC1* (AT3G22231), *AtMYB56* (AT5G17800), *AtGRDP2* (AT4G37900), *AtPELPK1* (AT5G09530), *OsCCR19* (Os09g0419200), *OsBNTB19* (Os09g0420900), *OsRNS4* (Os09g0537700), and *OsGA20OX1* (Os03g0856700).

## 5. Conclusions

In conclusion, the objective of the study was to validate the role of the maize ZmBES1/BZR1-9 transcription factor in regulating flowering time. The *ZmBES1/BZR1-9* gene was ectopically expressed in *Arabidopsis* and rice for phenotyping. Our findings demonstrate that ZmBES1/BZR1-9 is a positive regulator that promotes flowering via multiple photoperiod-mediated pathways, but it does not affect vegetative growth. Our results also suggest that the maize *ZmBES1/BZR1-9* gene can be used to improve the flowering period via its overexpression and provide a reference for further underlying roles of BES1/BZR1 in regulating growth and development in crops.

## Data Availability

All data are included in the article and Supplementary Files.

## Supplementary Materials

The following supporting information can be downloaded at: https://www.mdpi.com/article/10.3390/plants12162995/s1, Table S1: Primers used in the study; Table S2: The number of *E-box* and *BRRE* elements in the promoter region of the candidate gene.
